# Annotations

**Published:** 1900-06-30

**Authors:** 


### Annotations.

### The Responsibility for the Sickness Among our Troops.

It is impossible to read without distress, and, indeed, without the strongest sense of indignation, the revelations made in the *Times* of the 27th instant by Mr. Burdett-Coutts in regard to the hardships to which our sick soldiers in South Africa have been exposed—exposed, we fear, recklessly and almost wilfully, in consequence of the want of foresight displayed by the central administrative authorities, who ought to have known, what all experience has taught, that sickness would certainly throw a far more serious strain than wounds upon the medical organisation. No doubt we have now in South Africa a far larger force than was contemplated when the war began. But those who sent out the men ought surely to have had the forethought to send out the necessary equipment in addition, and it ought not to have been possible for such horrors to have come into existence as those which Mr. Burdett-Coutts details so graphically as having been seen with his own eyes. No one can doubt that the men on the spot were struggling manfully to meet the terrible strain of supplying the wants of the twenty thousand sick who now occupy the whole track of our army. More than half of these sick, we are told, are down with typhoid fever, and those who know best what care, what cleanliness, what constant attention are required to prevent infection spreading far and wide will recognise not only the terrible hardships to which our unfortunate soldiers are being exposed, but the serious danger which hangs over the whole expedition from the unchecked extension of this disease. We will not now enter into the details of the horrible neglect which Mr. Burdett-Coutts lays bare, but we will join with him in saying that the whole series of events which he describes ought to have been foreseen and provided for. That fever has always been the scourge of South Africa; that Bloemfontein is always a typhoid town; and that this town would of necessity become a point on which our troops would concentrate in large numbers was always known. And Bloemfontein is the very place where the things are happening which Mr. Burdett-Coutts describes. That many of the wounded have been well looked after we do not doubt. But who is responsible for this terrible want of foresight in regard to the sick? What we insist on is that the prevalence of typhoid ought to have been foreseen and provided for. Writing so far back as December in last year on this very subject of "War and Fever," we sounded a note of warning, and what was patent to us as journalists must have been perfectly obvious to those in authority. We said: "At present this country is in the midst of a great access of patriotism, and our brave fellows out in South Africa are with unfailing gallantry facing wounds and sudden death for glory and their country. But wounds are not the only penalties of warfare, and those who know most concerning fever pray the most earnestly that this war may soon be over."

### Rabies and its Lesson.

Mr. Long and the officers of the Board of Agriculture, who have worked so energetically for the suppression of rabies, are to be congratulated upon the practical success which has attended their efforts. For six months, we are told, no case of the disease has occurred in England and Wales, and although it may be premature to speak positively or to feel sure that there may not somewhere or other lie *perdu* an unreported case which may become the source of further trouble, we at least are warranted in expressing our appreciation of the steady and unflinching perseverance with which, in the face of unlimited abuse from persons who ought to have known better, Mr. Long has carried to a successful issue a carefully arranged plan for the suppression of this dangerous disease. While on the subject, and while congratulating all concerned on the success of their efforts, it may not be without interest to point out the moral which may be drawn regarding certain infectious diseases found among men, from the difficulty which has been experienced in suppressing rabies among dogs, notwithstanding the comparative simplicity of the latter problem. We say comparative simplicity with reason, for compared with the task of stamping out human infections that of controlling such a disease as rabies in dogs is a small affair. It is obvious that the "stamping out" problem is far simpler in the case of a disease which is infectious only by direct inoculation by the bite of an animal than where it can be transmitted in various ways, as is the case in many human infections; in a disease which can be isolated by the rough and ready plan of slaughtering the infected, than where it can only be done by imprisoning them in hospitals until infectivity ceases; in a disease the spread of which can be controlled by killing off all those which have been exposed even to a chance of becoming infected, than where, as among humans, nothing can be done until disease actually declares itself; and finally in a disease the infectivity of which can be minimised by muzzling, than where, as in human ailments, practically nothing can be done to prevent the transmission of stray infection. If, then, we consider the time and the trouble that it has taken

**ANNOTATIONS**—*continued.*

[illegible]o bring rabies well under control, notwithstanding all [illegible]hese advantages and facilities, and remember that even [illegible]ow it is only kept at bay by the maintenance of a [illegible]ystem of quarantine over all dogs coming from abroad, [illegible] system which would be absolutely impossible to [illegible]nforce in the case of any human disease, we do not see [illegible]uch to encourage the belief, which some have indulged [illegible]n, that by the persistent and consistent employment of [illegible]otification and isolation we shall ever be able to stamp [illegible]ut any one of the diseases to which man is subject. That we can exercise a very effective control over the spread of epidemics is true enough, but to "stamp out" a disease is another affair.

### The Society of Apothecaries of London.

The following important resolution has been passed by the Court of Assistants of the Society of Apothecaries of London, in regard to the assumption of the title of physician and surgeon by the Licentiates of the society: "The resolution passed by the General Medical Council at its recent session deciding not to employ the funds of the Council in defraying the expenses of a test case to determine whether the L.S.A. (1886) is or is not entitled to call himself physician and surgeon having been read, Resolved, that in view of the opinions expressed by eminent counsel in favour of the society's contention, and this Court considering that the decision of the General Medical Council ought not to operate to the prejudice of the Licentiates of the society, hereby authorises all Licentiates of the society qualified under the Medical Act, 1886, to call themselves physician and surgeon, and undertakes to defend them at the sole cost of the society from the legal consequences of their adopting those titles."

### The Modern Elevator.

We hear from America that the elevator "pilot," as he is called, is liable to suffer from serious heart trouble, due, it is said, to the nature of his calling. This is an interesting statement; for, although the condition does not seem to have attracted attention here, this may be due to the facts that on the one hand we have but few lifts running to the height they attain in the New York and Chicago sky-scrapers, and on the other that people here are not generally willing, if they can help it, to allow themselves to be whisked up and down at quite so high a speed as seems to be common on the other side. It is thought by some writers on the subject that the constant variation of atmospheric pressure experienced by those whose lives are passed swinging to and fro between heaven and earth may have much to do in producing cardiac derangement. This, however, seems somewhat open to question. The 100 ft. or so which is the extreme to which most English lifts reach is a mere trifle to that attained by many mountain "funiculars." It is to the strain upon the vascular mechanism caused by the repeated readjustments required on starting and stopping, especially when these movements are performed suddenly, that we must look for the cause of the evil complained of, and with the quickly acting multiplying mechanisms now in use there can be little doubt that the strain is sometimes a severe one. When a careless attendant allows the lift, and oneself within it, to practically drop through space, as sometimes happens, the sickening blow upon the solar plexus caused by the unopposed action of the abdominal muscles, and the fulness in the head which results from the vascular system failing to accommodate itself all at once to the new condition, are a far from pleasant experience, and one can easily see how injurious the constant repetition of the performance may be, especially in the case of an elderly man with vessels not quite so elastic and accommodating as they should be. We know many people who, though quite willing to go up, entirely decline to go down. The old-fashioned plunger lift, with its ponderous chains, was no doubt sometimes a trial to patience; but the modern highflyer, which shoots one up like a rocket and drops one like a ball, abolishing for the time all those relations of our internals which depend on gravitation, is a trial in other directions which are much more important.

### The Standardisation of Drugs.

It sounds like an axiom to say that in ordering drugs one ought to know their exact strength. We fear, however, that we often are far from doing so; in fact, it is the uncertainty which pervades the whole question of dosage that leads to the reiterated demands for some system of standardisation, some system in accordance with which we might prescribe according to the effect to be produced, rather than the amount to be swallowed. The matter, however, is not quite so simple as it may seem. With some drugs, of course, the active principles of which are capable of being correctly determined by chemical means, one has but to fix a standard strength and stick to it. But there are substances which cannot be so dealt with, and others which depend for their efficacy on several alkaloids which occur in varying proportions. The ideal method then is physiological standardisation—the testing of the drug upon an animal before it is given to man. But, as is pointed out by Dr. Rice, of New York, in the *American Journal of Pharmacy*, unless a uniform method were agreed upon and accepted by the different makers there would be but little likelihood of their results agreeing; and he asks, not inappropriately, Who is to standardise the standardisers? There is no question that the difficulty is a real one, and we can hardly doubt that one at least of the reasons why medical men so often order proprietary preparations of some of the more variable drugs is the idea and the hope that by so doing they are likely to obtain a more uniform effect than they would do by merely prescribing according to the Pharmacopœia. To the physician the actual strength of a drug does not matter. He learns his dosages by practice, not by reference to a manual, and all he asks is that the strength of the preparation shall be always the same. Whether he gets what he asks is another affair, but it is the hope of so doing that leads him so often to order fancy preparations, or to append the maker's name in his prescription, both of which are things the doing of which goes rather against his principles, and both of which, we fear, only emphasise still further the unfortunate tendency of all trades to drift into the hands of the great traders. It is hard upon the smaller and really dispensing chemists.

